# Accuracy Improvement Method Based on Characteristic Database Classification for IMRT Dose Prediction in Cervical Cancer: Scientifically Training Data Selection

**DOI:** 10.3389/fonc.2022.808580

**Published:** 2022-03-03

**Authors:** Yiru Peng, Yaoying Liu, Zhaocai Chen, Gaolong Zhang, Changsheng Ma, Shouping Xu, Yong Yin

**Affiliations:** ^1^ Shandong Cancer Hospital and Institute, Shandong First Medical University and Shandong Academy of Medical Sciences, Jinan, China; ^2^ School of Physics, Beihang University, Beijing, China; ^3^ Manteia Technologies Co., Ltd, Xiamen, China; ^4^ National Cancer Center/Cancer Hospital, Chinese Academy of Medical Sciences and Peking Union Medical College, Beijing, China

**Keywords:** deep learning, radiotherapy, cervical cancer, database classification, IMRT, dose prediction

## Abstract

**Purpose:**

Consistent training and testing datasets can lead to good performance for deep learning (DL) models. However, a large high-quality training dataset for unusual clinical scenarios is usually not easy to collect. The work aims to find optimal training data collection strategies for DL-based dose prediction models.

**Materials and Methods:**

A total of 325 clinically approved cervical IMRT plans were utilized. We designed comparison experiments to investigate the impact of (1) beam angles, (2) the number of beams, and (3) patient position for DL dose prediction models. In addition, a novel geometry-based beam mask generation method was proposed to provide beam setting information in the model training process. What is more, we proposed a new training strategy named “full-database pre-trained strategy”.

**Results:**

The model trained with a homogeneous dataset with the same beam settings achieved the best performance [mean prediction errors of planning target volume (PTV), bladder, and rectum: 0.29 ± 0.15%, 3.1 ± 2.55%, and 3.15 ± 1.69%] compared with that trained with large mixed beam setting plans (mean errors of PTV, bladder, and rectum: 0.8 ± 0.14%, 5.03 ± 2.2%, and 4.45 ± 1.4%). A homogeneous dataset is more accessible to train an accurate dose prediction model (mean errors of PTV, bladder and rectum: 2.2 ± 0.15%, 5 ± 2.1%, and 3.23 ± 1.53%) than a non-homogeneous one (mean errors of PTV, bladder and rectum: 2.55 ± 0.12%, 6.33 ± 2.46%, and 4.76 ± 2.91%) without other processing approaches. The added beam mask can constantly improve the model performance, especially for datasets with different beam settings (mean errors of PTV, bladder, and rectum improved from 0.8 ± 0.14%, 5.03 ± 2.2%, and 4.45 ± 1.4% to 0.29 ± 0.15%, 3.1 ± 2.55%, and 3.15 ± 1.69%).

**Conclusions:**

A consistent dataset is recommended to form a patient-specific IMRT dose prediction model. When a consistent dataset is not accessible to collect, a large dataset with different beam angles and a training model with beam information can also get a relatively good model. The full-database pre-trained strategies can rapidly form an accuracy model from a pre-trained model. The proposed beam mask can effectively improve the model performance. Our study may be helpful for further dose prediction studies in terms of training strategies or database establishment.

## 1 Introduction

In recent decades, with the emergence and development of advanced radiotherapy (RT) planning and delivery techniques such as intensity-modulated radiation therapy (IMRT) and volumetric-modulated arc therapy (VMAT), the quality of radiotherapy plans has drastically improved with better target volume dose coverage and normal tissue sparing (1, 2). However, there are still many obstacles in current clinical planning practice. The planning objectives or constraints generated using population-based standard clinical protocols may lead to sub-optimal or even infeasible plan quality for specific patients (3, 4). In addition, the trial-and-error planning process (5, 6) highly depends on the skills and experience of the planners. It is time-consuming and labor-intensive, and results in significant variations in plan quality (7, 8).

Many novel methods have been developed and introduced into clinics for improving treatment plan efficiency, quality, and consistency (9, 10). The knowledge-based planning (KBP) (7, 11, 12) generates the reference plans for a new patient using a dose-volume histogram (DVH) model trained from historical treatment plans (13, 14). However, the clinical application of this method is limited because of the unsatisfying output as only one-dimensional DVH, rather than the 3D dose distributions (15) and the need for manual interventions, such as planning target volume (PTV)-organ at risk (OAR) distance and PTV’s length (16, 17). In recent years, with the rapid advances in computational power, the deep learning (DL) technique has drawn significant attention and become research hotspots in many fields (11, 18). The DL-based dose prediction method, which takes advantage of CNN’s automatic features extraction ability, can build the relationship between anatomical information and the dose distribution of a patient. Many studies have shown the significant success of various DL methods in predicting 3D dose distributions for different treatment sites and delivery methods (3, 19, 20).

At the current stage, most of the DL dose prediction models require a large number of consistent and high-quality training datasets and the applications are limited to the cases that have the same characteristics, such as the same beam settings (numbers and angles) and the patient positions (majority is at supine position). However, a large high-quality training dataset is usually not easy to collect for unusual clinical scenarios, such as unique beam arrangements. The impact of the quality and quantity of training database on the DL model performance has been explored in other studies (21, 22), but only limited to the impact of the homogeneity and size of the database, and no other method about improving IMRT dose prediction accuracy, or making a suggestion for how to make use of a sizable non-homogeneous dataset. This work explores optimal training data collection strategies for DL-based dose prediction models. To the best of our knowledge, there have not been efforts reported in this direction. In this work, we designed comparison experiments to investigate the impact of (1) beam angles, (2) the number of beams, and (3) patient position for DL dose prediction models. In addition, a novel geometry-based beam mask generation method was proposed to provide beam setting information in the model training process, which makes the model more accurate. What is more, we proposed a new training strategy named “full-database pre-trained strategy”. Our new training strategy might rapidly use a heterogeneous dataset to form a patient-specific dose prediction model.

## 2 Methods and Materials

### 2.1 Patient Data and Treatment Planning

A total of 325 clinically approved and delivered cervical cancer IMRT plans were retrospectively selected for the DL model training and testing. All plan contours of the PTV and OARs have been checked by experienced radiation oncologists. The prescription dose of PTV cases was 50.4 Gy in 28 fractions. The treatment plan used different beam settings in beam numbers (7 or 9 beams, all coplanar), beam angles, and patient treatment positions (supine and prone) according to the patient’s anatomy features. All the treatments were planned using the Eclipse treatment planning system with the Anisotropic Analytical Algorithm (AAA). All dose constraints of IMRT for cervical cancer are based on Quantitative Analyses of Normal Tissue Effects in the Clinic (QUANTEC) data.

### 2.2 Data Preparation

The purpose of the data preparation process is to ensure that the DL model could correctly process the mapping and transformation between anatomical information and the dose distribution of a patient.

**Figure 1:**
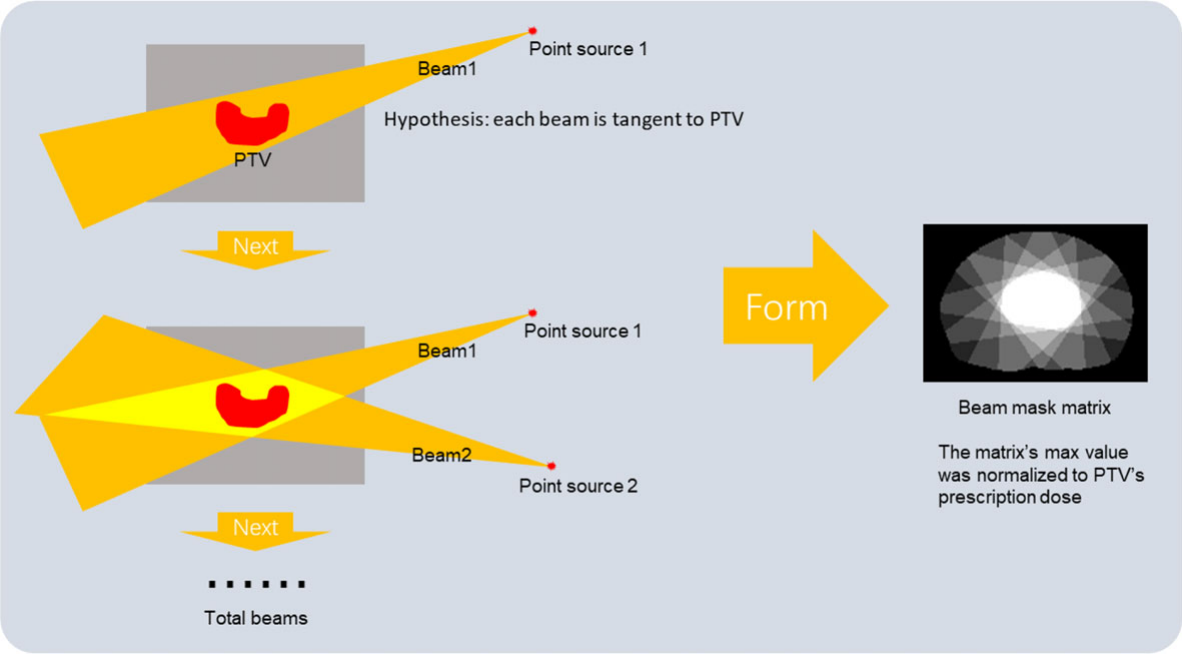
The generation process of beam mask.

In IMRT treatment, the beam setting can significantly influence patients’ dose distribution. So, in addition to the image and contouring mask as model inputs, we also proposed a novel generation method of beam mask to feed the extra beam setting information (beam angles and beam numbers) to the DL model training, which is in a logic of clinical scenarios to predict dose distribution. The beam setting information was first extracted from the RT plan file. We hypothesized that each beam source is a point source, and all the beams were tangent to PTV through the isocenter, which matches the clinical logic and can present the beam information to the model. This work generated a beam mask by assigning 1 to pixels within the beam path while 0 outside the beam. All the beam masks were then summed and rescaled to the maximum of 50.4 Gy to match the prescription dose for PTV and reduce the burden of DL model fitting (a form of normalization of the model’s input and output data: making beam mask’s value more similar to the training dose distribution, reducing the parameter update steps of model training). Our new beam mask production method was an analytical algorithm based on the geometric method. Compared with the existing dose engine-based and TPS-based beam mask generation methods (19), our method requires fewer computing resources and is much easier to realize. The details of making a beam mask are shown in **Figure 1**.

**Figure 2:**
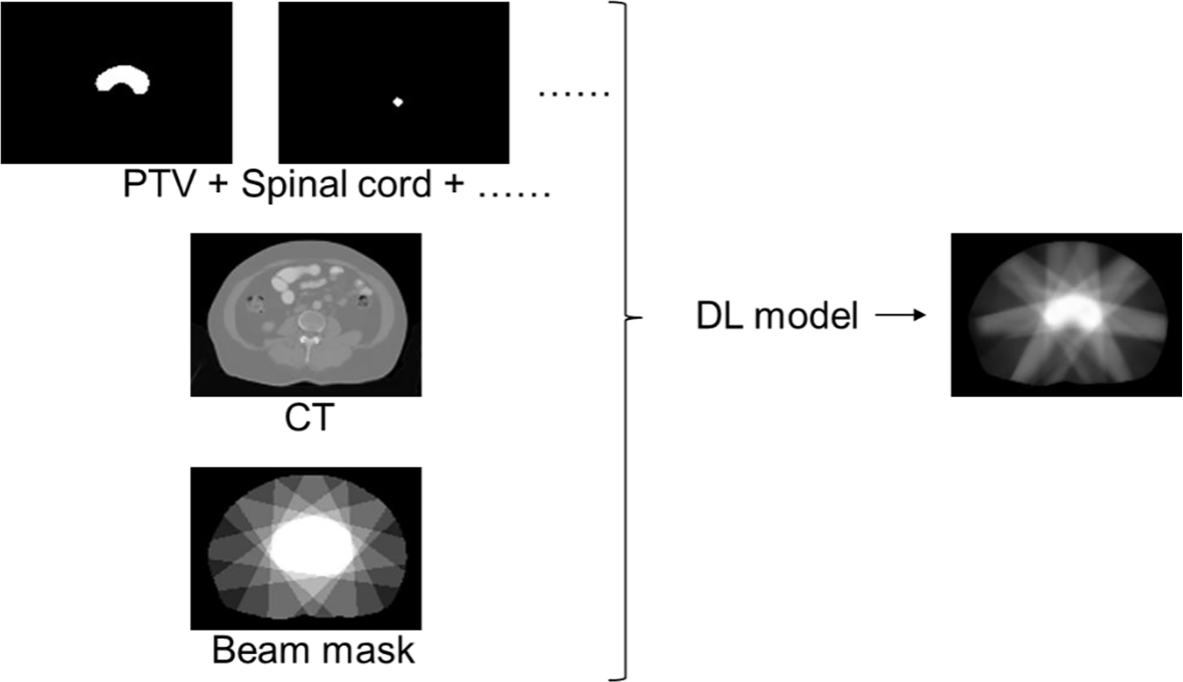
Data preparation diagram.

We first extracted a 3D matrix of 512 × 512 × 100 ( ± 30) for each plan from CT images. It was then normalized to have a mean value of 0 and a variance of 1. A binary mask was generated for each ROI, with 1 for voxels inside the contour and 0 for those outside the contour. A 3D dose array was also extracted from the RT dose file. For fast dose calculation purposes, all the clinical plans were calculated using a 2.5-mm dose grid, which is different from the resolution of the original CT images [1 ( ± 0.2) mm × 1 ( ± 0.2) mm × 5 mm]. Therefore, the images and contouring masks were resampled to match the solution of the dose matrix. The images, contouring masks, and the dose matrix were then rigidly registered and used for DL model training. **Figure 2** shows the diagram of the DL model input and output.

Python codes did the data pre-processing for all preparation processes. Python packages such as NumPy and pydicom were used to conserve the raw data to the “npy” format.

### 2.3 Model Architecture and Training Method

A 3D Dense U-Net CNN model was built. The model input data were a multi-channel 3D matrix, including CT images, ROI contouring masks for bladder, body, left femoral head (Femoral-Head-L), right femoral head (Femoral-Head-R), PTV, rectum, spinal cord, and beam mask (if the beam is added as a feature). The model output was the corresponding predicted 3D dose matrix. It shows the model structure and training method’s details in **Appendix-1** (Model architecture and training method).

### 2.4 Experiment Design

Four experiments were designed to explore the influence of dataset characteristics in IMRT dose prediction mode training. The first series of experiments explored the total usage of a sizable non-homogeneous dataset. The second, third, and fourth experiments explore the influence of unification of beam angles, beam numbers, and patient position, which are essential factors that can influence dose distribution in IMRT plans.

#### 2.4.1 Full-Database Experiments With Pre-Trained Strategy

**Figure 3:**
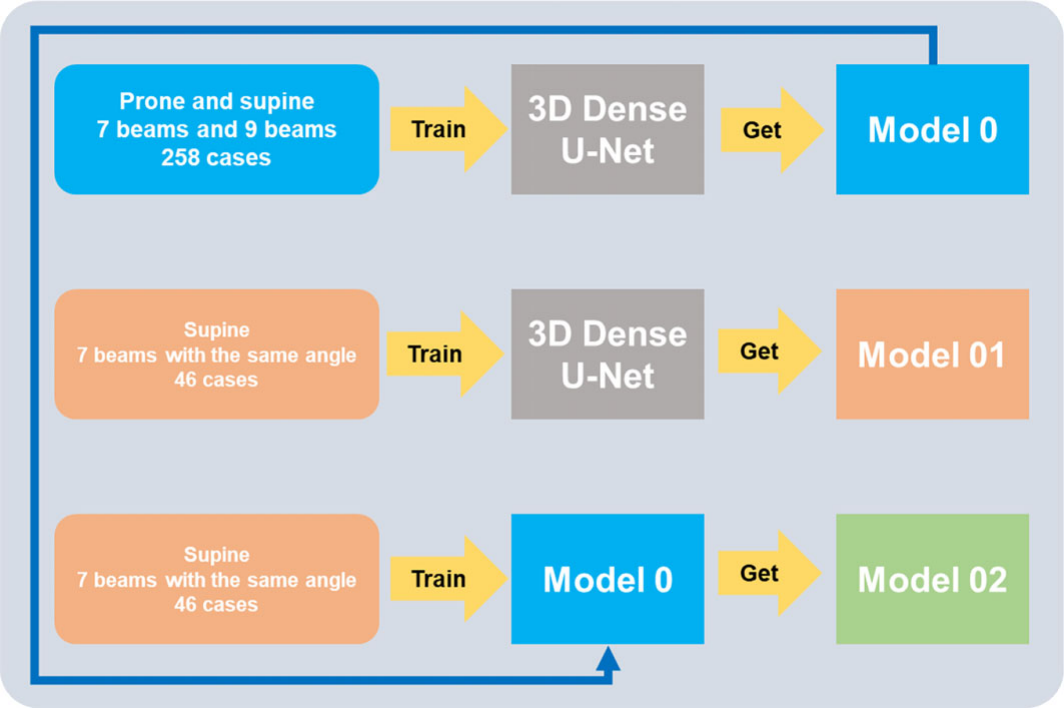
Experiments to prove a full-database pre-trained strategy.

A total of 9 cases were randomly selected as testing cases, which were all treated with seven beams of 0°, 50°, 100°, 150°, 210°, 260°, and 310° at the supine position. As shown in **Figure 3**, we designed three different experiments. In Experiment 1-1, Model 0 was trained by taking the full usage of 258 training cases from total cases number 325, including different beam numbers or angles and different patient positions. In Experiment 1-2, Model 01 was trained using 46 patients with the same beam setting as the saved testing cases. In Experiment 1-3, Model 02 was first trained by reserving the model weight from Model 0 and training the selected 46 patients. The prediction errors of the three models were calculated and compared. Then, we used the best model training strategy in the following experiments.

#### 2.4.2 Influence of Unified Beam Angles

To investigate the influence of uniform beam angles in the training dataset and the extra model input of beam mask on the performance of the dose prediction model, we conducted four comparative experiments. The same testing cases as in the previous section were used in these experiments. In Experiment 2-1, we selected the 46 cases with the same beam configuration in the testing cases. In addition, the beam mask was added as an extra input channel in the training process. In Experiment 2-2, the same training cases as in Experiment 2-1 but without the beam masks. In Experiment 2-3, we selected 213 cases with seven beams as training data and used beam masks in the training process. Experiment 2-3 aimed to study whether a significant number of cases in the training dataset with different beam angles can improve the model performance. In Experiment 2-4, the training cases were the same as Experiment 2-3, but Experiment 2-4 had the added beam masks in the training process.

#### 2.4.3 Influence of Unified Beam Numbers

To investigate the influence of uniform beam numbers in the training dataset and the extra model input of beam mask on the performance of the dose prediction model, we conducted four comparative experiments.

We selected 12 testing cases treated with nine beams. Experiments 3-1 and 3-2 were to compare beam performance using either the training data of the same beam number (i.e., nine beams) or a more extensive training dataset with the mixed numbers of beams (i.e., 9 and 7 beams). Because of the limitation of 9-beam cases (21 cases), training and testing cases had different beam angle distributions. The beam masks were used for both experiments. In Experiment 3-3, the model was trained with only 7-beam cases to investigate model performance in the testing cases with different beams. In Experiment 3-4, the training cases were the same as Experiment 3-2 but without beam masks as input.

#### 2.4.4 Influence of Unified Positioning

Most of the cervical patients in our department were scanned with the supine position, but there are still some cases with the prone position. We designed four comparison experiments using different training datasets to investigate the strategy for optimal selection of DL model training data, particularly for the clinical scenario with fewer cases such as prone position.

We selected 13 test cases scanned in the prone position. In Experiment 4-1, we selected a total of 258 training cases with both prone position and supine position, while in Experiment 4-2, only the 45 prone cases were used for model training. The beam masks were added as model input for both experiments to provide beam information to help the model predict the accurate doses. For comparison, in Experiment 4-3, we removed the beam masks from Experiment 4-2. In Experiment 4-4, the model was trained with only the supine position cases. Due to the dataset’s size of prone cases being small (45 cases), training and testing cases have different beam angle distributions.

### 2.5 Model Performance Evaluation Method

The percentage of errors (δ*Di*) was calculated to evaluate all experiments. The formula of the percentage of errors was:


$$\delta Di=\frac{\left|Di_{Ground-truth}-Di_{predicted}\right|}{prescription\;dose}\times 100\%$$

We calculated δ*Di* (a total of 14 DVH indices) of D_95_, D_90_, D_50_, D_max_, and D_mean_ of PTV; V_30_, D_max_, and D_mean_ of bladder, rectum; and D_max_ of spinal cord and femoral heads.

We selected the best model in each series of experiments by synthetically considering the dose-prediction errors. We measured the model by the 14 DVH indices: If the model gets the maximum optimal dose indices (% of best prediction is the number of optimal DVH indices/the total number of DVH indices), the model is the best in the group of experiments.

## 3 Results

### 3.1 Full-Database Pre-Trained Strategy Experiments

**Figure 4:**
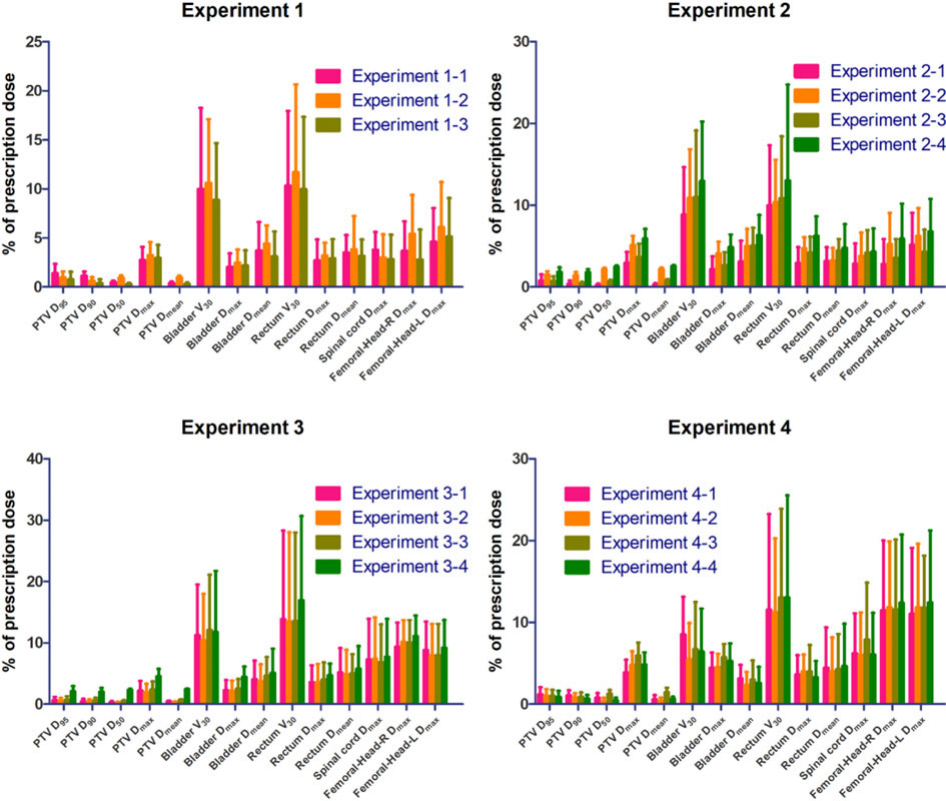
The absolute percentage errors for ROIs.

Under these three experiments (Experiments 1-1 vs. 1-2 vs. 1-3), on the one hand, the errors of the PTV D_95_ decreased from 1.39 ± 0.95% for Experiment 1-1 to 0.96 ± 0.60% for Experiment 1-2, to 0.76 ± 0.79% for Experiment 1-3. On the other hand, for the considered OARs, the errors of the mean doses were reduced from 2.63 ± 1.63% and 3.80 ± 2.33% in Experiment 1-2 to 2.33 ± 1.67% and 3.18 ± 2.07% in Experiment 1-1, and then to 2.18 ± 1.67% and 3.10 ± 2.24% in Experiment 1-3. The summary of prediction errors of each experiment is shown in **Figure 4**.

### 3.2 Experiments of Beam Settings

**Figure 4** shows the average predicted percentage dose errors of the mean and maximum doses in PTV and OARs. The model trained with a small database but with the same beam setting and beam information (Experiment 2-1 with 46 cases) outperformed the model trained with an extensive database with different beam settings (Experiments 2-3 and 2-4 with 213 cases) and a small database with the same beam setting but no beam information (Experiment 2-2 with 46 cases). With the added beam mask information, Experiment 2-1 yielded the best performance with the lowest mean dose errors across all the organs (2.18 ± 1.67%), followed by Experiments 2-2, 2-3, and 2-4 with mean dose errors of 3.21 ± 1.85%, 2.70 ± 1.43%, and 3.85 ± 2.13%, respectively. Similarly, Experiment 2-1 also had the lowest average errors of predicted max dose (3.11 ± 2.24%), while Experiments 2-2, 2-3, and 2-4 were 4.90 ± 2.16%, 3.73 ± 2.10%, and 5.70 ± 2.50%, respectively.

### 3.3 Experiments on the Number of Beams

For the model trained with a small database with the same beam number (i.e., nine beams) and beam information (Experiment 3-1), when adding different beam numbers, i.e., the model trained with an extensive database with mixed beam number (i.e., 9 or 7 beams) (Experiment 3-2). The predicted errors of the bladder V30 in Experiment 3-2 were 0.9%, 1.7%, and 1.4%, which were lower than the predicted values in Experiments 3-1, 3-3, and 3-4, respectively (**Appendix-3-Table 3**). For the rest of the plotted DVH metrics (PTV D_95_, PTV D_90_, PTV D_50_, PTV D_max_, PTV D_mean_, Rectum D_max_, Rectum D_mean_, Bladder D_max_, Bladder D_mean_, Rectum V_30_, and Left femoral head D_max_), Experiment 3-2 had the slightest prediction error. In particular, Experiment 3-1 outperformed other experiment groups for the PTV D_50_, which reduced to 0.21%.

### 3.4 Experiments on Treatment Positions

For a model trained in a database with mixed treatment positions (i.e., prone or supine position) and beam information (Experiment 4-1), when we removed the supine position data, only in the model trained in the prone position database with beam information (Experiment 4-2) were the predicted errors decreased in most of the organs, especially the bladder and rectum; the D_mean_ decreased by 0.8% and 0.5%, respectively. When the beam mask was removed from Experiment 4-2, the model trained only with the prone position database (Experiment 4-3), the prediction errors were significantly improved.

### 3.5 The Best Model in Each Series of Experiments

We evaluated our model’s performance using the “Model performance evaluation method” in **Section 2.5** and the “% of best prediction”. It shows that the best model in a series of experiments is in Experiment 1-3, Experiment 2-1, Experiment 3-2, or Experiment 4-2, separately (shown in **Appendix-2-Table 1**). Each model’s prediction errors are shown in **Appendix-3-Tables 1–4**.

**Appendix-3-Figures 1** and **4** show the results of the testing patients in each group of experiments with the three-dimensional dose distribution predicted by each group of experiments and the corresponding DVHs.

## 4 Discussion

This study aimed to analyze the impact of classifying training databases on the performance of DL models for dose prediction in the framework of radiotherapy for cervical cancer. For this purpose, we set 3 groups of experiments to study the influence of a uniform training database with beam angles, beam numbers, and patient positions on the accuracy of a prediction model. We also proposed a new beam information mask generation method, which can quickly and accurately learn beam angle information and convert the beam settings into beam masks to achieve the best model performance. What is more, we created a new “full-database pre-trained strategy”, which makes full use of a wide range of databases more effectively to build and obtain more accurate prediction models.

Some conclusions can be drawn across four groups of experiments, and the conclusions may be helpful in the IMRT dose prediction model training process and database establishment. First, a homogeneous dataset is more accessible to train an accurate dose prediction model than a non-homogeneous one without other processing approaches. This conclusion can be drawn from Experiment 2-2 vs. Experiment 2-4. These two experiments are both without additional processing. We found that the model trained with the 46-size homogeneous dataset (all cases have the same beam settings) performed better than the other model trained with the 213-size non-homogeneous dataset (the cases have different beam settings). Two model performance details can be seen from **Figure 4**, **Appendix-3-Figure 1**, and **Appendix-3-Table 2**.

Since the non-homogeneous dataset with different beam settings may cause a suboptimal model, we added a beam mask to provide beam setting information in training processing and tried to make a prediction model that establishes the relationship between beam settings and dose distribution. Moreover, the second conclusion can be that beam information can make the non-homogeneous models perform well. The conclusion may be drawn from Experiment 2-3 vs. Experiment 2-4 and Experiment 3-2 vs. Experiment 3-4. Visual comparisons are shown in **Appendix-3-Figure 1**. When the beam mask was added, the predicted dose accuracy was improved in global and PTV areas. Furthermore, when the beam mask was added, a non-homogeneous dataset’s performance was close to a homogeneous one (Experiment 2-1 vs. Experiment 2-3, Experiment 3-1 vs. Experiment 3-2). The beam mask made the homogeneous dataset perform better (Experiment 2-1 vs. Experiment 2-2). The conclusion that beam masks can make the mixed beam setting models perform well was following clinical logic. The beam setting can significantly influence patients’ dose distribution in the planning design process. So, using the beam mask to present beam information to the model, following the planning design logic, can make dose prediction more accurate.

Besides adding beam mask, another method to make usage of the non-homogeneous dataset is the “full-database pre-trained strategy”. In the second conclusion, we know that beam mask can make the non-homogeneous dataset’s performance close to the homogeneous one, so a pair of experiments were made (1-1 vs. 1-2); we found that an extensive non-homogeneous dataset’s model (Model 0) performance beat a small homogeneous dataset’s (Model 01) in almost all evaluation indicators when the beam information was added in the training processing. However, another question arises: the large dataset (256 cases) training process is time-consuming, costing several days (on an RTX 3090 GPU). So, another experiment 1-3 was made: using a 46-size homogeneous dataset to continue training Model 0 (256-size non-homogeneous dataset’s model) to get Model 02. We found that Model 02’s performance was better than that of Model 0, and the time cost of 46 cases refining Model 0 was within an hour. The third conclusion can be that when the beam information is involved in IMRT dose prediction model training, using a small homogeneous dataset to refine a sizable non-homogeneous dataset’s model can get a good performance model, and the time cost can be much reduced compared with training a model from the beginning by a large dataset that combines the homogeneous and non-homogeneous dataset (an hour vs. several days).

Another important finding in our research is that the dataset size dramatically influences the accuracy of IMRT dose prediction model. When the beam mask involved the experiments, different performances among the homogeneous and non-homogeneous datasets could be seen in some experiments (2-1 vs. 2-3), but not in other experiments (3-1 vs. 3-2, 4-1 vs. 4-2). When analyzing the above phenomenon and dataset features, we focused on the sizes of the homogeneous dataset across the above experiments. Experiment 2-1 has a 46-size homogeneous dataset (the same beam settings), but a total number of 9 beam cases and prone cases, which were with the non-unified beam angles, only have 21 and 45. The level of homogeneity was lower than Experiment 2-1, and the dataset size was relatively small, so the advantage of the homogeneity dataset in Experiments 3-1 and 4-2 was not evident as 2-1. From the above discussion, an excess conclusion can be drawn that the beam angle unification is more effective than beam numbers and patient positions.

Some shortcomings were listed below, which can be renovated in future related work. Our analysis shows that the homogeneous dataset may be advantageous compared to the non-homogeneous one when the homogeneity and dataset size level are enough. We have not collected enough nine beam cases and prone cases with the same beam settings to show the advantage of the homogeneity in beam numbers and patient positions. Future research may use a larger dataset to explore more characteristics of homogeneous and non-homogeneous datasets. Our conclusion showed that a homogeneous dataset could improve model accuracy, but the largest dataset with the same beam settings has only 46 cases. The model with the largest homogenous dataset got the best prediction errors (Experiment 2-1), which proved that the homogenous dataset might benefit the model’s accuracy again. The lack of homogeneous datasets led to our prediction errors not being as good as the current study. Nevertheless, the conclusions in our research, such as a homogeneous dataset, suggested that providing beam settings in the training process might make future research a better dose prediction model. Advance computer technology may improve the experiment performance. Our study focused on the dataset with a relatively conventional U-net-like model. The novel structure such as attention-gate and the multi-stage network could be involved in the model architecture, making prediction more accurate. The “full-database pre-trained strategy” in our study used the homogeneous dataset for continuing to train a pre-trained model, which focused more on concepts than methods. Meta-learning technology aimed to use small data to refine a pre-trained model and improve performance. Meta-learning might make the “full-database pre-trained strategy” perform better. Further efforts can expand the investigations onto different tumor types with different treatment techniques.

## 5 Conclusion

This study designed different experiments to explore the influence of different clinical scenarios in IMRT dose prediction model training, such as various beam angles, the number of beams, and different patient positions. A homogeneous dataset is more accessible to train an accurate dose prediction model than a non-homogeneous one without other processing approaches. The beam angles of the dataset cases can significantly influence IMRT dose prediction accuracy. In the IMRT model training process, the beam information is suggested to be included. In the IMRT dose prediction dataset collection process, a compatible size dataset with the same beam angles is recommended. If the homogeneous data are hard to collect, training a model using a non-homogeneous dataset combined with beam information can also get a relatively accurate model.

Besides, in our study, a novel training strategy and beam information array generation method were proposed. The “full-database pre-trained strategies” used a small size dataset to re-train the model trained by a large dataset to form a specific model, which can get an accurate model and reduce the time-consuming training for the model. The proposed geometric-based beam mask generation method can effectively provide beam setting information and improve the model performance.

Our study may be helpful for further dose prediction studies in terms of training strategies or database establishment.

## Data Availability Statement

The raw data supporting the conclusions of this article will be made available by the authors, without undue reservation.

## Author Contributions

YP: Experiment design, data collection and article writing. YL: Experiment design, code implementation and article writing. ZC: Technical support. GZ: The article modification. CM: Data collection. SX: Experiment design and article modification. YY: Data collection and article modification. All authors contributed to the article and approved the submitted version.

## Funding

The work was supported by the Medical Big Data AI R&D Project (2019MBD-043), the National Natural Science Foundation of China (Grant No. 82072094), the Natural Science Foundation of Shandong Province (Grant No. ZR2019LZL017), and the Taishan Scholars Project of Shandong Province (Grant No. ts201712098).

## Conflict of Interest

Manteia Technologies Co., Ltd employed author ZC.

The remaining authors declare that the research was conducted in the absence of any commercial or financial relationships that could be construed as a potential conflict of interest.

## Publisher’s Note

All claims expressed in this article are solely those of the authors and do not necessarily represent those of their affiliated organizations, or those of the publisher, the editors and the reviewers. Any product that may be evaluated in this article, or claim that may be made by its manufacturer, is not guaranteed or endorsed by the publisher.
