# Supplementary material for: Accuracy Improvement Method Based on Characteristic Database Classification for IMRT Dose Prediction in Cervical Cancer: Scientifically Training Data Selection
Source: Front Oncol. 2022 Mar 3;12:808580. doi: 10.3389/fonc.2022.808580 (PMC8927290; doi:10.3389/fonc.2022.808580)
Supplement: Supplementary file 1 [file DataSheet_1.pdf]

Appendix-1

Model architecture and training method

A 3D Dense U-Net CNN model was built (as shown in Appendix-1-Fig. 1). The U-Net model architecture consists of down-sampling, up-sampling and concatenation structures across the bottleneck, which allows the model to learn the high-, middle-, and low-level features. Because of the outstanding feature extraction and feature restore ability, the U-net structure has been widely used in end-to-end matrix mapping. The Dense U-Net structure combines the characteristic of U-Net and DenseNet, preserves the U-Net's down-sampling and up-sampling structures, and combines densely connected layers within each hierarchical level of U-Net. Every hierarchical level of Dense U-Net preserves all features from previous levels, allowing feature reuse and propagation. Compared with the 2D Dense U-Net, the 3D Dense U-Net captures features between different slices. Since it directly learns the input matrix's 3D information, 3D Dense U-Net has proved effective at spatial feature extraction and mapping transformation.

The model input data were a multi-channel matrix, including CT images, beam mask (if the beam is added as a feature), and ROI contouring masks for bladder, body, left femoral head, right femoral head, PTV, rectum, spinal cord, respectively. The model output was the corresponding dose matrix. Due to the GPU RAM limitations, the study used a patch-training strategy. For the model to learn more information from all spatial orientations, we selected a rather big patch size in all dimensions: the patch size of 48×112×96 used for training was randomly selected from the entire 3D dose matrix. The 3D Dense U-NET model was down-sampled four times using max-pooling (kernel shape 2×2×2) and up-sampled four times using deconvolution (kernel shape 2×2×2, channels = 100). The down-sampling reduced the initial input matrix shape from (48×112×96) to (3×7×6), leading the model to learn both local and global features. The up-sampling restored the matrix shape to (48×112×96). The final convolution layer formed a single channel matrix as the output matrix. The size of each convolution kernel was 3×3×3. Zero padding was used in convolution, and each convolution layer had 16 channels. Every convolution layer was followed by batch normalization, concatenation, and a RELU activation. We used the Adam optimizer with MSE loss and a batch size of 4. The learning rate decayed from 10^{-4} to 10^{-6} during model training, and training was terminated when the loss function and learning rate stabilized. The deep learning framework was TensorFlow and Keras. The model was trained and tested using an RTX Titan GPU with 24 GB of dedicated memory.

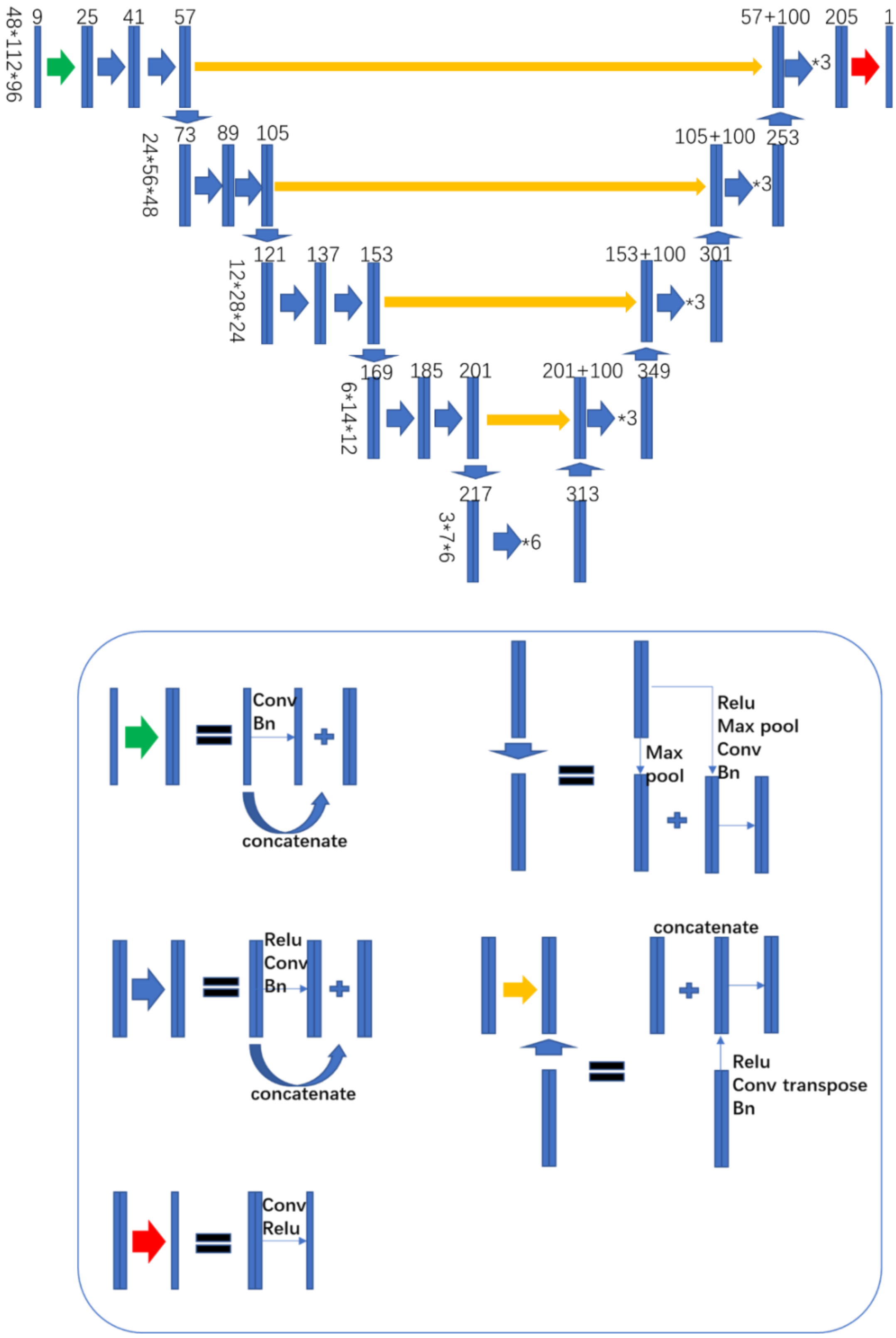

Appendix-1-Fig. 1. 3D Dense U-NET architecture
