# Supplementary material for: Accuracy Improvement Method Based on Characteristic Database Classification for IMRT Dose Prediction in Cervical Cancer: Scientifically Training Data Selection
Source: Front Oncol. 2022 Mar 3;12:808580. doi: 10.3389/fonc.2022.808580 (PMC8927290; doi:10.3389/fonc.2022.808580)
Supplement: Supplementary file 2 [file DataSheet_2.pdf]

Appendix-2

Appendix-2-Tab. 1. The experiment details and the model performance

Training data / Experiments	Case numbers	Beam mask	Unified beam angles	Using pre-trained model	Results / Testing cases	% of best prediction	Best model
Experiment 1-1	258	√	×	-	7 beams & same beam setting	28.6%	
Experiment 1-2	45	√	√	×		0%	
Experiment 1-3	***45***	**√**	**√**	**√**		***71.4%***	**√**
Training data / Experiments	Case numbers	Beam mask	Unified beam angles	7 beams	Results / Testing cases	% of best prediction	Best model
Experiment 2-1	***46***	**√**	**√**	**√**	7 beams & same beam setting	***85.7%***	**√**
Experiment 2-2	46	×	√	√		0%	
Experiment 2-3	213	√	×	√		14.3%	
Experiment 2-4	213	×	×	√		0%	
Training data / Experiments	Case numbers	Beam mask	7 beams	9 beams	Results / Testing cases	% of best prediction	Best model
Experiment 3-1	21	√	×	√	9 beams	7.1%	
Experiment 3-2	***251***	**√**	**√**	**√**		***85.8%***	**√**
Experiment 3-3	230	√	√	×		7.1%	
Experiment 3-4	251	×	√	√		0%	
Training data / Experiments	Case numbers	Beam mask	Prone	Supine	Results / Testing cases	% of best prediction	Best model
Experiment 4-1	258	√	√	√	Prone	28.6%	
Experiment 4-2	***45***	**√**	**√**	**×**		***50.0%***	**√**
Experiment 4-3	45	×	√	×		0%	
Experiment 4-4	213	√	×	√		21.4%	
