# Supplementary material for: Accuracy Improvement Method Based on Characteristic Database Classification for IMRT Dose Prediction in Cervical Cancer: Scientifically Training Data Selection
Source: Front Oncol. 2022 Mar 3;12:808580. doi: 10.3389/fonc.2022.808580 (PMC8927290; doi:10.3389/fonc.2022.808580)
Supplement: Supplementary file 3 [file DataSheet_3.pdf]

Appendix-3

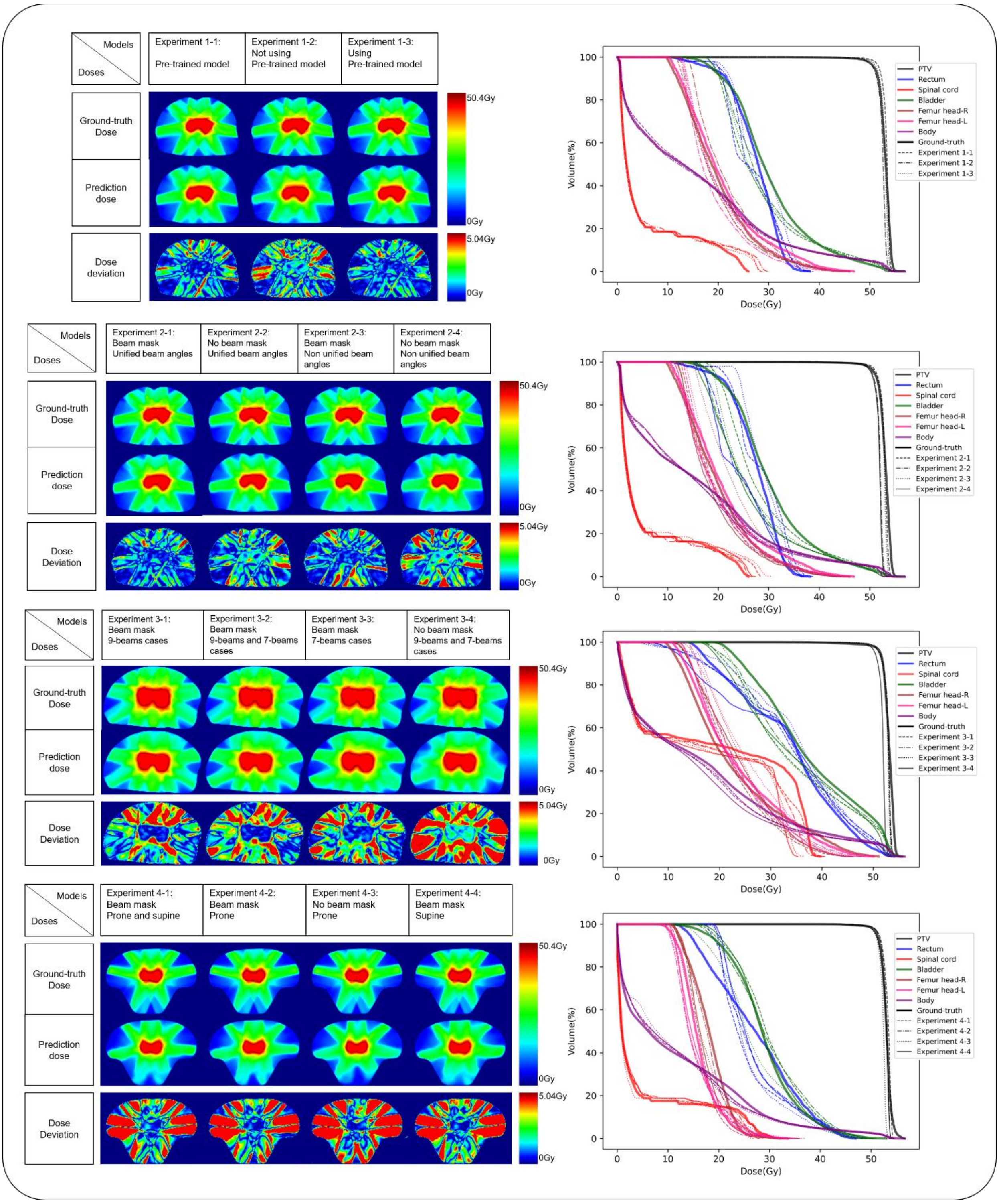

Appendix-3-Fig. 1. The different model’s performance

Appendix-3-Tab. 1. Absolute errors for DVH metrics in the first series of experiments

Mean absolute errors for DVH metrics(mean ± SD)					
			Clinical	Predicted	|Errors| (%)
PTV	D_{95} (Gy)	Experiment 1-1	50.90±0.47	51.60±0.14	1.39±0.95
		Experiment 1-2	50.90±0.47	50.64±0.14	0.96±0.60
		Experiment 1-3	50.90±0.47	51.15±0.08	0.76±0.79
	D_{90}(Gy)	Experiment 1-1	51.64±0.20	52.22±0.07	1.14±0.42
		Experiment 1-2	51.64±0.20	51.36±0.10	0.61±0.39
		Experiment 1-3	51.64±0.20	51.80±0.06	0.38±0.40
	D_{50} (Gy)	Experiment 1-1	53.20±0.09	53.43±0.04	0.46±0.18
		Experiment 1-2	53.20±0.09	52.74±0.07	0.91±0.25
		Experiment 1-3	53.20±0.09	53.06±0.06	0.27±0.13
	D_{max}(Gy)	Experiment 1-1	56.46±0.55	55.08±0.32	2.72±1.35
		Experiment 1-2	56.46±0.55	54.82±0.30	3.23±1.35
		Experiment 1-3	56.46±0.55	54.96±0.28	2.95±1.34
	D_{mean} (Gy)	Experiment 1-1	52.98±0.03	53.17±0.09	0.39±0.16
		Experiment 1-2	52.98±0.03	52.49±0.09	0.96±0.17
		Experiment 1-3	52.98±0.03	52.83±0.06	0.29±0.15
Bladder	V_{30} (%)	Experiment 1-1	58.19±9.51	52.53±11.47	9.98±8.26
		Experiment 1-2	58.19±9.51	49.81±9.72	10.60±6.49
		Experiment 1-3	58.19±9.51	55.26±10.42	8.86±5.79
	D_{max}(Gy)	Experiment 1-1	55.10±1.07	54.18±0.48	2.04±1.38
		Experiment 1-2	55.10±1.07	53.86±0.72	2.46±1.36
		Experiment 1-3	55.10±1.07	54.06±0.60	2.17±1.56
	D_{mean} (Gy)	Experiment 1-1	33.55±2.17	32.75±2.44	3.71±2.90
		Experiment 1-2	33.55±2.17	32.07±2.26	4.41±1.85
		Experiment 1-3	33.55±2.17	33.17±2.26	3.10±2.55
Rectum	V_{30} (%)	Experiment 1-1	53.21±14.76	51.72±13.14	10.30±7.63
		Experiment 1-2	53.21±14.76	47.67±17.53	11.70±8.94
		Experiment 1-3	53.21±14.76	55.93±12.27	9.95±7.38
	D_{max}(Gy)	Experiment 1-1	50.35±5.07	48.99±4.80	2.69±2.14
		Experiment 1-2	50.35±5.07	48.74±4.72	3.19±1.32
		Experiment 1-3	50.35±5.07	49.01±4.64	2.91±1.96
	D_{mean} (Gy)	Experiment 1-1	31.60±2.77	30.60±2.49	3.48±1.81
		Experiment 1-2	31.60±2.77	30.23±3.22	3.82±3.41
		Experiment 1-3	31.60±2.77	31.28±2.40	3.15±1.69
Spinal cord	D_{max} (Gy)	Experiment 1-1	29.62±3.87	29.99±4.36	3.78±1.82
		Experiment 1-2	29.62±3.87	29.49±3.40	2.99±2.37
		Experiment 1-3	29.62±3.87	30.12±4.27	2.82±2.50
Femoral-Head-R	D_{max} (Gy)	Experiment 1-1	43.81±3.95	43.04±2.67	3.67±3.02
		Experiment 1-2	43.81±3.95	41.44±4.23	5.41±3.95
		Experiment 1-3	43.81±3.95	42.77±3.34	2.78±3.08
	D_{max} (Gy)	Experiment 1-1	45.86±3.90	44.12±4.56	4.59±3.45
		Experiment 1-2	45.86±3.90	42.97±3.68	6.08±4.61

Femoral-Head-L		Experiment 1-3	45.86±3.90	43.49±4.46	5.13±3.93

Appendix-3-Tab. 2. Absolute errors for DVH metrics in the second series of experiments

Mean absolute errors for DVH metrics(mean ± SD)					
			Clinical	Predicted	|Errors| (%)
PTV	D_{95} (Gy)	Experiment 2-1	50.90±0.47	51.15±0.08	0.76±0.79
		Experiment 2-2	50.90±0.47	50.27±0.14	1.49±0.43
		Experiment 2-3	50.90±0.47	50.88±0.09	0.71±0.58
		Experiment 2-4	50.90±0.47	50.03±0.20	1.83±0.57
	D_{90}(Gy)	Experiment 2-1	51.64±0.20	51.80±0.06	0.38±0.40
		Experiment 2-2	51.64±0.20	50.93±0.05	1.42±0.41
		Experiment 2-3	51.64±0.20	51.55±0.05	0.42±0.19
		Experiment 2-4	51.64±0.20	50.74±0.10	1.79±0.38
	D_{50} (Gy)	Experiment 2-1	53.20±0.09	53.06±0.06	0.27±0.13
		Experiment 2-2	53.20±0.09	52.10±0.05	2.16±0.17
		Experiment 2-3	53.20±0.09	52.84±0.05	0.70±0.18
		Experiment 2-4	53.20±0.09	51.96±0.04	2.45±0.17
	D_{max} (Gy)	Experiment 2-1	56.46±0.55	54.96±0.28	2.95±1.34
		Experiment 2-2	56.46±0.55	53.87±0.27	5.12±1.11
		Experiment 2-3	56.46±0.55	54.65±0.68	3.62±1.66
		Experiment 2-4	56.46±0.55	53.48±0.49	5.90±1.21
	D_{mean} (Gy)	Experiment 2-1	52.98±0.03	52.83±0.06	0.29±0.15
		Experiment 2-2	52.98±0.03	51.86±0.08	2.20±0.15
		Experiment 2-3	52.98±0.03	52.57±0.06	0.80±0.14
		Experiment 2-4	52.98±0.03	51.69±0.07	2.55±0.12
Bladder	V_{30} (%)	Experiment 2-1	58.19±9.51	55.26±10.42	8.86±5.79
		Experiment 2-2	58.19±9.51	49.91±10.18	10.88±5.94
		Experiment 2-3	58.19±9.51	51.90±11.49	10.97±8.16
		Experiment 2-4	58.19±9.51	46.57±10.27	12.94±7.27
	D_{max}(Gy)	Experiment 2-1	55.10±1.07	54.06±0.60	2.17±1.56
		Experiment 2-2	55.10±1.07	53.04±0.54	4.07±1.46
		Experiment 2-3	55.10±1.07	53.78±0.62	2.62±1.62
		Experiment 2-4	55.10±1.07	52.65±0.42	4.86±1.54
	D_{mean} (Gy)	Experiment 2-1	33.55±2.17	33.17±2.26	3.10±2.55
		Experiment 2-2	33.55±2.17	31.70±2.21	5.00±2.10
		Experiment 2-3	33.55±2.17	31.77±2.34	5.03±2.20
		Experiment 2-4	33.55±2.17	30.80±2.09	6.33±2.46
Rectum	V_{30} (%)	Experiment 2-1	53.21±14.76	55.93±12.27	9.95±7.38
		Experiment 2-2	53.21±14.76	49.64±15.73	10.35±5.20
		Experiment 2-3	53.21±14.76	52.40±14.69	10.85±7.58
		Experiment 2-4	53.21±14.76	42.13±18.28	13.00±11.74
	D_{max}(Gy)	Experiment 2-1	50.35±5.07	49.01±4.64	2.91±1.96
		Experiment 2-2	50.35±5.07	47.97±4.85	4.71±1.36

		Experiment 2-3	50.35±5.07	48.24±4.71	4.19±1.94
		Experiment 2-4	50.35±5.07	47.20±4.97	6.24±2.39
	D_{mean} (Gy)	Experiment 2-1	31.60±2.77	31.28±2.40	3.15±1.69
		Experiment 2-2	31.60±2.77	30.44±3.16	3.23±1.53
		Experiment 2-3	31.60±2.77	30.27±1.65	4.45±1.40
		Experiment 2-4	31.60±2.77	29.33±3.55	4.76±2.91
Spinal cord	D_{max} (Gy)	Experiment 2-1	29.62±3.87	30.12±4.27	2.82±2.50
		Experiment 2-2	29.62±3.87	29.71±4.86	3.79±2.87
		Experiment 2-3	29.62±3.87	31.03±4.25	4.16±2.77
		Experiment 2-4	29.62±3.87	29.71±4.74	4.30±2.85
Femoral-Head-R	D_{max} (Gy)	Experiment 2-1	43.81±3.95	42.77±3.34	2.78±3.08
		Experiment 2-2	43.81±3.95	41.35±3.31	5.25±3.80
		Experiment 2-3	43.81±3.95	43.60±2.64	3.55±2.31
		Experiment 2-4	43.81±3.95	40.94±2.97	5.89±4.29
Femoral-Head-L	D_{max} (Gy)	Experiment 2-1	45.86±3.90	43.49±4.46	5.13±3.93
		Experiment 2-2	45.86±3.90	42.82±3.73	6.21±3.44
		Experiment 2-3	45.86±3.90	44.06±3.85	4.29±2.71
		Experiment 2-4	45.86±3.90	42.50±3.88	6.76±4.00

Appendix-3-Tab. 3. Absolute errors for DVH metrics in the third series of experiments

Mean absolute errors for DVH metrics(mean ± SD)					
			Clinical	Predicted	|Errors| (%)
PTV	D_{95} (Gy)	Experiment 3-1	51.24±0.35	51.05±0.17	0.65±0.53
		Experiment 3-2	51.24±0.35	51.17±0.13	0.50±0.51
		Experiment 3-3	51.24±0.35	50.95±0.15	0.71±0.59
		Experiment 3-4	51.24±0.35	50.18±0.19	2.10±0.84
	D_{90}(Gy)	Experiment 3-1	51.85±0.26	51.72±0.12	0.47±0.40
		Experiment 3-2	51.85±0.26	51.81±0.10	0.39±0.39
		Experiment 3-3	51.85±0.26	51.62±0.12	0.61±0.44
		Experiment 3-4	51.85±0.26	50.83±0.12	2.03±0.61
	D_{50} (Gy)	Experiment 3-1	53.17±0.05	53.01±0.08	0.31±0.18
		Experiment 3-2	53.17±0.05	53.07±0.09	0.21±0.17
		Experiment 3-3	53.17±0.05	52.90±0.06	0.53±0.13
		Experiment 3-4	53.17±0.05	51.98±0.06	2.35±0.15
	D_{max} (Gy)	Experiment 3-1	56.02±0.50	55.00±0.64	2.19±1.58
		Experiment 3-2	56.02±0.50	55.15±0.57	1.91±1.42
		Experiment 3-3	56.02±0.50	54.91±0.57	2.36±1.34
		Experiment 3-4	56.02±0.50	53.73±0.38	4.53±1.21
	D_{mean} (Gy)	Experiment 3-1	52.99±0.02	52.76±0.06	0.45±0.13
		Experiment 3-2	52.99±0.02	52.84±0.06	0.30±0.13
		Experiment 3-3	52.99±0.02	52.65±0.05	0.67±0.11
		Experiment 3-4	52.99±0.02	51.76±0.05	2.44±0.10
	V_{30} (%)	Experiment 3-1	62.46±19.22	56.11±17.23	11.23±8.27
		Experiment 3-2	62.46±19.22	57.38±16.34	10.38±7.61

		Experiment 3-3	62.46±19.22	54.87±17.18	12.07±9.05
		Experiment 3-4	62.46±19.22	52.95±17.51	11.73±9.98
Bladder	D_{max}(Gy)	Experiment 3-1	55.11±1.04	54.07±0.70	2.25±1.66
		Experiment 3-2	55.11±1.04	54.09±0.72	2.15±1.68
		Experiment 3-3	55.11±1.04	53.86±0.65	2.51±1.57
		Experiment 3-4	55.11±1.04	52.86±0.68	4.45±1.68
	D_{mean} (Gy)	Experiment 3-1	34.94±4.27	33.84±4.16	4.06±3.05
		Experiment 3-2	34.94±4.27	34.15±3.99	3.70±2.80
		Experiment 3-3	34.94±4.27	33.56±4.16	4.62±3.06
		Experiment 3-4	34.94±4.27	32.81±4.29	5.09±3.94
Rectum	V_{30} (%)	Experiment 3-1	68.17±18.60	60.57±16.54	13.84±14.46
		Experiment 3-2	68.17±18.60	66.74±16.82	13.47±14.55
		Experiment 3-3	68.17±18.60	62.39±20.20	13.53±14.44
		Experiment 3-4	68.17±18.60	57.40±18.35	16.94±13.71
	D_{max}(Gy)	Experiment 3-1	52.09±4.33	50.98±4.37	3.56±2.77
		Experiment 3-2	52.09±4.33	51.08±4.35	3.54±2.97
		Experiment 3-3	52.09±4.33	50.81±4.33	4.00±2.80
		Experiment 3-4	52.09±4.33	50.10±4.07	4.68±1.93
	D_{mean} (Gy)	Experiment 3-1	34.90±5.17	33.79±4.26	5.15±3.99
		Experiment 3-2	34.90±5.17	34.89±4.48	4.84±4.01
		Experiment 3-3	34.90±5.17	34.48±4.62	5.01±3.11
		Experiment 3-4	34.90±5.17	33.38±4.74	5.76±3.71
Spinal cord	D_{max} (Gy)	Experiment 3-1	33.62±4.97	1.74±1.48	7.27±6.65
		Experiment 3-2	33.66±4.97	1.72±1.68	7.38±6.75
		Experiment 3-3	33.43±3.93	1.61±1.62	6.79±6.22
		Experiment 3-4	32.44±3.56	1.40±1.64	7.72±6.20
Femoral-Head-R	D_{max} (Gy)	Experiment 3-1	44.37±6.30	43.43±3.26	9.33±3.97
		Experiment 3-2	44.37±6.30	43.51±3.36	10.14±3.56
		Experiment 3-3	44.37±6.30	43.89±3.18	10.04±3.66
		Experiment 3-4	44.37±6.30	43.14±3.13	11.10±3.36
Femoral-Head-L	D_{max} (Gy)	Experiment 3-1	47.55±4.36	45.94±3.16	8.82±4.65
		Experiment 3-2	47.55±4.36	46.62±3.10	7.95±5.12
		Experiment 3-3	47.55±4.36	46.80±2.89	7.97±5.11
		Experiment 3-4	47.55±4.36	45.29±3.00	9.18±4.55

Appendix-3-Tab. 4. Absolute errors for DVH metrics in the fourth series of experiments

Mean absolute errors for DVH metrics(mean ± SD)					
			Clinical	Predicted	|Errors| (%)
	D_{95} (Gy)	Experiment 4-1	50.89±0.48	51.42±0.32	1.17±0.90
		Experiment 4-2	50.89±0.48	51.30±0.32	1.01±0.82
		Experiment 4-3	50.89±0.48	50.55±0.35	1.01±0.74
		Experiment 4-4	50.89±0.48	50.86±0.40	0.88±0.75
		Experiment 4-1	51.59±0.29	52.09±0.23	1.06±0.63

	D_{90}(Gy)	Experiment 4-2	51.59±0.29	51.91±0.22	0.80±0.53
		Experiment 4-3	51.59±0.29	51.21±0.23	0.88±0.57
		Experiment 4-4	51.59±0.29	51.56±0.27	0.66±0.48
PTV	D_{50} (Gy)	Experiment 4-1	53.00±0.27	53.40±0.05	0.79±0.56
		Experiment 4-2	53.00±0.27	53.06±0.06	0.35±0.43
		Experiment 4-3	53.00±0.27	52.37±0.07	1.28±0.45
		Experiment 4-4	53.00±0.27	52.95±0.07	0.45±0.34
	D_{max} (Gy)	Experiment 4-1	56.95±0.68	55.01±0.27	3.86±1.56
		Experiment 4-2	56.95±0.68	54.55±0.33	4.76±1.71
		Experiment 4-3	56.95±0.68	53.95±0.22	5.95±1.57
		Experiment 4-4	56.95±0.68	54.52±0.24	4.82±1.51
	D_{mean} (Gy)	Experiment 4-1	52.86±0.27	53.14±0.10	0.57±0.55
		Experiment 4-2	52.86±0.27	52.82±0.10	0.42±0.38
		Experiment 4-3	52.86±0.27	52.12±0.12	1.45±0.56
		Experiment 4-4	52.86±0.27	52.67±0.13	0.63±0.28
Bladder	V_{30} (%)	Experiment 4-1	48.12±18.41	53.23±22.36	8.53±4.60
		Experiment 4-2	48.12±18.41	49.81±21.87	5.48±4.44
		Experiment 4-3	48.12±18.41	43.12±22.61	6.71±5.77
		Experiment 4-4	48.12±18.41	50.84±22.74	6.42±5.25
	D_{max}(Gy)	Experiment 4-1	54.84±1.35	52.60±1.38	4.44±1.86
		Experiment 4-2	54.84±1.35	52.57±1.14	4.50±1.63
		Experiment 4-3	54.84±1.35	51.95±1.08	5.73±1.62
		Experiment 4-4	54.84±1.35	52.19±1.70	5.27±2.15
	D_{mean} (Gy)	Experiment 4-1	31.03±4.36	32.05±4.87	3.12±1.69
		Experiment 4-2	31.03±4.36	31.55±5.00	2.33±1.61
		Experiment 4-3	31.03±4.36	29.97±5.36	2.97±2.38
		Experiment 4-4	31.03±4.36	31.66±4.87	2.58±1.97
Rectum	V_{30} (%)	Experiment 4-1	47.73±20.73	46.51±27.09	11.54±11.70
		Experiment 4-2	47.73±20.73	45.57±24.37	11.25±9.20
		Experiment 4-3	47.73±20.73	44.36±27.33	13.03±10.85
		Experiment 4-4	47.73±20.73	46.74±27.06	13.02±12.51
	D_{max}(Gy)	Experiment 4-1	51.24±3.59	51.20±2.30	3.61±2.37
		Experiment 4-2	51.24±3.59	50.72±2.34	3.99±2.08
		Experiment 4-3	51.24±3.59	49.98±3.09	3.97±3.26
		Experiment 4-4	51.24±3.59	50.79±2.51	3.26±2.00
	D_{mean} (Gy)	Experiment 4-1	30.12±5.15	30.87±6.15	4.46±4.91
		Experiment 4-2	30.12±5.15	30.71±5.45	3.98±4.18
		Experiment 4-3	30.12±5.15	30.20±6.01	4.26±4.31
		Experiment 4-4	30.12±5.15	30.96±5.96	4.67±5.17
Spinal cord	D_{max} (Gy)	Experiment 4-1	30.61±7.32	28.81±5.11	6.22±4.87
		Experiment 4-2	30.61±7.32	28.52±5.05	5.99±5.22
		Experiment 4-3	30.61±7.32	26.83±4.92	7.91±6.95
		Experiment 4-4	30.61±7.32	29.45±4.68	6.05±5.10
		Experiment 4-1	41.31±4.93	36.62±3.09	11.48±8.53

Femoral-Head-R	D_{max} (Gy)	Experiment 4-2	41.31±4.93	36.74±2.99	11.85±8.06
		Experiment 4-3	41.31±4.93	36.18±2.71	11.57±8.56
		Experiment 4-4	41.31±4.93	36.73±2.89	12.38±8.34
Femoral-Head-L	D_{max} (Gy)	Experiment 4-1	40.94±7.08	39.11±4.29	11.04±8.07
		Experiment 4-2	40.94±7.08	38.592±4.28	11.83±7.77
		Experiment 4-3	40.94±7.08	37.77±3.18	11.78±6.35
		Experiment 4-4	40.94±7.08	39.09±3.91	12.43±8.80
